# Modulation of G-protein activation, calcium currents and opioid receptor phosphorylation by the pH-dependent antinociceptive agonist NFEPP

**DOI:** 10.3389/fnmol.2023.1171855

**Published:** 2023-05-12

**Authors:** Melih Özgür Celik, Viola Seitz, Fatih Yergöz, Sandeep Dembla, Nina Kathleen Blum, Stefan Schulz, Christoph Stein

**Affiliations:** ^1^Department of Experimental Anesthesiology, Charité – Universitätsmedizin Berlin, Corporate Member of Freie Universität Berlin and Humboldt-Universität zu Berlin, Berlin, Germany; ^2^Department of Pharmacology, Universitätsklinikum Jena, Jena, Germany

**Keywords:** NFEPP, mu opioid receptor (MOR), VDCC, fentanyl, pain

## Abstract

N-(3-fluoro-1-phenethylpiperidine-4-yl)-N-phenyl propionamide is a newly-designed pain killer selectively activating G-protein-coupled mu-opioid receptors (MOR) in acidic injured tissues, and therefore devoid of central side effects which are typically elicited at normal pH values in healthy tissues. However, the neuronal mechanisms underlying NFEPP’s antinociceptive effects were not examined in detail so far. Voltage-dependent Ca^2+^ channels (VDCCs) in nociceptive neurons play a major role in the generation and inhibition of pain. In this study, we focused on the effects of NFEPP on calcium currents in rat dorsal root ganglion (DRG) neurons. The inhibitory role of the G-protein subunits G_*i/o*_ and Gβγ on VDCCs was investigated using the blockers pertussis toxin and gallein, respectively. GTPγS binding, calcium signals and MOR phosphorylation were also investigated. All experiments were performed at acidic and normal pH values using NFEPP in comparison to the conventional opioid agonist fentanyl. At low pH, NFEPP produced more efficient G-protein activation in transfected HEK293 cells and significantly reduced VDCCs in depolarized DRG neurons. The latter effect was mediated by Gβγ subunits, and NFEPP-mediated MOR phosphorylation was pH-dependent. Fentanyl’s responses were not affected by pH changes. Our data indicate that NFEPP-induced MOR signaling is more effective at low pH and that the inhibition of calcium channels in DRG neurons underlies NFEPP’s antinociceptive actions.

## Introduction

Opioids are the strongest painkillers to date. Clinically used opioid drugs (e.g., morphine, fentanyl) mainly target the mu opioid receptor (MOR), a G-protein coupled receptor (GPCR). The activation of MORs in the peripheral (PNS) and central nervous system (CNS) produces strong pain relief, but their activation in the CNS also results in serious side effects including addiction and respiratory depression (Imam et al., 2018). Recently, our group designed a new MOR agonist, (±)-N-(3-fluoro-1-phenethylpiperidine-4-yl)-N-phenyl propionamide (NFEPP), with a lower acid dissociation constant (pK_*a*_) (Spahn et al., 2017; Rosas et al., 2019; Lešnik et al., 2020; Ray et al., 2020). NFEPP selectively activated peripheral MORs in inflamed tissues at low pH values (common in most painful injuries) whereas a conventional opioid (fentanyl) was equally active at both low and normal pH values (Spahn et al., 2017; Rodriguez-Gaztelumendi et al., 2018; Stein, 2018; Del Vecchio et al., 2019; Jiménez-Vargas et al., 2022). Apparently, the decreased pK_*a*_ value precludes the protonation of NFEPP in healthy tissues (pH > 7.35; e.g., in brain) but facilitates its protonation in injured/inflamed microenvironments (low pH, high concentrations of protons). Consistent with the notion that the protonation of opioid ligands is typically required for the binding to opioid receptors (Lešnik et al., 2020), NFEPP was active only in injured tissues.

Targeting opioid receptors in the PNS at the site of injury produces strong analgesia, as previously demonstrated: (i) locally applied opioids relieve severe clinical pain and a large proportion of the analgesic effects of systemically administered opioids is mediated by opioid receptors in the PNS (Weibel et al., 2013; Jagla et al., 2014; Wei et al., 2014; Machelska and Celik, 2018; Sun et al., 2019; Martínez and Abalo, 2020; Jin et al., 2022); (ii) input from peripheral sensory neurons is essential in numerous pain syndromes (Berta et al., 2017; Sexton et al., 2018); (iii) upon injury, MOR expression is upregulated in peripheral sensory neurons and the perineurial barrier is disrupted, eventually increasing accessibility of the drugs’ target [reviewed in Machelska and Celik (2018), Jeske (2019)].

So far, the cellular mechanisms underlying NFEPP-induced antinociception were not examined in detail. In particular, it is not known how NFEPP-induced MOR activation is translated into inhibition of neuronal excitability, an essential prerequisite for analgesia. At the level of sensory neurons, voltage dependent Ca^2+^ channels (VDCC) play a major role in the generation and inhibition of pain. These channels regulate the neuronal excitation by allowing Ca^2+^ influx (Lu and Ikeda, 2016; Weiss and Zamponi, 2021). Following the activation of MOR and dissociation of the heterotrimeric G-protein complex, Gβγ subunits displace the beta subunit of VDCC, thereby decreasing the channel’s activation rate, leading to a dormant state where the channel is less likely to open and allow ions to flow through. This results in a decreased excitability of the sensory neuron [reviewed in Proft and Weiss (2015), Lu and Ikeda (2016), Weiss and Zamponi (2021)]. Finally, MOR is phosphorylated and internalized (Mann et al., 2015; Machelska and Celik, 2018; Jeske, 2019).

In this study, we investigated the effects of NFEPP at acidic and physiological pH in comparison to the conventional agonist fentanyl on G-protein activation, on endogenous VDCCs and on MOR phosphorylation.

## Materials and methods

### Animals

Animal experiments were approved by the State animal care committee (Landesamt für Gesundheit und Soziales, Berlin, Germany) and followed the ARRIVE guidelines (Kilkenny et al., 2010). Male Wistar rats (125–150 g, 5–6 weeks old; Janvier, Le Genest-Saint-Isle, France) were housed in groups of 1–2 per cage in a 12 h light/dark cycle, lined with ground corncob bedding and with free access to standard laboratory rodent chow and tap water. Room temperature was 22 ± 0.5^°^C and humidity was 60–65%. We investigated males only because the preclinical literature suggests that MOR signaling is not gender- or sex-dependent (Huhn et al., 2018; Barpujari et al., 2020).

### Chemicals and drugs

N-(3-fluoro-1-phenethylpiperidine-4-yl)-N-phenyl propionamide was synthesized and provided by ASCA GmbH (Berlin, Germany) as described in Spahn et al. (2017). Fentanyl citrate (F3886) and naloxone hydrochloride (NLX; N7758), were purchased from Sigma-Aldrich (Taufkirchen, Germany). Pertussis toxin (PTX) and gallein were purchased from Tocris (Wiesbaden-Nordenstadt, Germany). Radioactively labeled GTPγS kits were purchased from Perkin Elmer (Freiburg, Germany).

Chemicals used for membrane preparation and [^35^S]-guanosine-5’-O-(3-thio)-triphosphate ([^35^S]-GTPγS) binding: Trizma^§^ Preset Crystals (Sigma-Aldrich, Taufkirchen, Germany), bovine serum albumin (BSA), dithiothreitol (DTT), GDP, [^35^S]-GTPγS, GTPγS, optiphase HISAFE 3 (Perkin Elmer, Waltham, MA, USA); HEM G-protein buffer: 8 mM HEPES, 8 mM EPPS, 8 mM MES, 100 mM NaCl, 0.2 mM EGTA, 5 mM MgCl_2_; all from Sigma-Aldrich (Taufkirchen, Germany). Chemicals used for dissociation and culture of sensory neurons: Dulbecco’s Modified Eagles Medium (DMEM)/HAM’s F-12 medium (Biochrom F4815, Berlin, Germany), Penicillin (10,000 U), Streptomycin (10 mg/ml), 1.25% Collagenase (Sigma-Aldrich, Taufkirchen, Germany), 2.5% Trypsin (Sigma-Aldrich, Taufkirchen, Germany), acridine orange/propidium iodide (Logos, Villeneuve, France).

Chemicals used for patch clamp experiments: Extracellular buffer (ECS) (10 mM CaCl_2_⋅6H_2_O, 130 mM TEA-Cl_2_, 5 mM HEPES, 25 mM d-glucose; adjusted to pH 7.4 or 6.5; all from Sigma-Aldrich, Taufkirchen, Germany). Intracellular buffer (ICS) (105 mM CsCl, 2.5 mM MgCl_2_, 40 mM HEPES, 10 mM EGTA, 2 mM Mg-ATP, 0.5 mM GTP, 5 mM d-glucose; adjusted to pH 7.4 or 6.5; all from Sigma-Aldrich, Taufkirchen, Germany).

Chemicals used for calcium imaging: Fura 2 AM (Thermo-Fisher, Hennigsdorf, Germany). Extracellular solution: 130 mM NaCl, 3 KCl mM, 2 mM CaCl_2_, 0.6 mM MgCl_2_, 1 mM NaHCO_3_ 10 mM HEPES, 5 mM D glucose (adjusted to pH 7.4 or 6.5). High K+ solution contained: 61 NaCl mM, 75 KCl mM, 2 CaCl_2_ mM, 0.6 MgCl_2_ mM, 1 NaHCO_3_ mM, 10 Hepes mM, 5 D-glucose mM (adjusted to pH 7.4 or 6.5); all from Sigma-Aldrich (Taufkirchen, Germany).

Chemicals used for MOR phosphorylation experiments: The phosphosite-specific MOR antibodies against pT370-MOR (7TM0319B), pS375-MOR (7TM0319C), pT376-MOR (7TM0319D), and pT379-MOR (7TM0319E) as well as the phosphorylation-independent antibodies against np-MOR (7TM0319N) and rabbit polyclonal anti-HA antibodies (7TM000HA) were provided by 7TM Antibodies (Jena, Germany). [D-Ala^2^, N-MePhe^4^, Gly-ol]-enkephalin (DAMGO) acetate salt and dimethyl sulfoxide (DMSO) were purchased from Sigma-Aldrich (Darmstadt, Germany). Fentanyl citrate was obtained from Rotexmedica (Trittau, Germany).

### Culture and transfection of human embryonic kidney cells

Human embryonic kidney (HEK293) cells (German Collection of microorganisms and Cell Cultures, Braunschweig, Germany; RRID:CVCL_0045) were maintained in full medium containing DMEM media supplemented with fetal bovine serum (Biochrom, Berlin, Germany), penicillin (100 U/ml, Biochrom) and streptomycin (100 μg/ml, Biochrom) with or without geneticin (G418, 100 μg/ml, Biochrom), in 5% CO_2_ at 37^°^C as described before (Spahn et al., 2017). Cells were passaged 1:3–1:20 every second to third day from p8 and p28 depending on confluence.

For MOR transfection, wild-type HEK293 cells were plated on 30 mm diameter plastic culture dishes coated with poly-L-lysine 24 h before transfection. Confluent HEK293 cells (70–90%) were transfected with 1 μg per 200 μl transfection mix of each plasmid containing the different cDNAs using “X-tremeGENE HP DNA Transfection Reagent” (Roche; Mannheim, Germany) following the supplier’s recommendations. The plasmid containing the cDNA encoding the FLAG-epitope-tagged rat MOR (oprm1, NM 013071.2) in pcDNA™ 3.1 vector with geneticin resistance gene was provided by Prof. Christian Zöllner (University Hamburg, Germany). The cells were later grown in T175 flasks to have enough membranes for further experiment.

### Membrane preparation

Membrane fractions were prepared from transfected HEK293 cells as described previously (Zöllner et al., 2003). The cells were grown in 175 cm^2^ tissue culture flasks to approximately 90% confluence. Cells were then washed with Tris buffer (50 mM, Trizma preset crystals, pH 7.4; Sigma Aldrich, Darmstadt, Germany), harvested with a scraper, homogenized using a mechanical disperser (Dispergierstation T8.10, IKA-Werke, Staufen, Germany) at maximum speed for 10 s and centrifuged at 42 K × g for 20 min at 4^°^C (Avanti JXN-26 ultracentrifuge, Beckmann Coulter, Krefeld, Germany). Cellular pellets including membranes with embedded and anchored proteins were then resuspended in Tris buffer for washing to separate them from cytosolic components by homogenization and centrifugation at the same settings. Supernatants were discarded and the pellets were stored at -80^°^C. On the day of usage, the pellets were thawed on ice in Tris buffer and homogenized. Total protein concentrations were determined according to the Bradford method (Bradford, 1976) (Bio-Rad Laboratories GmbH, München, Germany) and homogenates were split according to the number of conditions tested in respective assay buffers.

### Isolation and culture of sensory neurons

Dorsal root ganglions were harvested from naïve male Wistar rats following their sacrifice by an overdose of isoflurane (5%) (AbbVie, Wiesbaden, Germany). The thoracic and lumbar spinal regions were exposed and all DRGs were collected in a working medium (DMEM/HAM’s F-12 without penicillin/streptomycin). A total of 1.25% collagenase was added just before incubation. Following their incubation at 37^°^C for 60 min, DRGs were washed three times with PBS and incubated in the working medium digestive solution with trypsin for 10 min at 37^°^C. Thereafter, the tissue was further triturated using plastic pipette tips and filtered through a 40 μl filter. The filtrate was centrifuged and the pellet was resuspended in 1 ml culture medium (DMEM/HAM’s F12 supplemented with 1% penicillin/streptomycin and 10% horse serum). Neurons were then seeded onto poly-L-lysine coated plastic culture dishes (35 mm) and placed in an incubator (5% CO_2_ at 37^°^C). One hour later, 2 ml of culture medium were added to the cell culture and neurons were incubated for 24–48 h until patch clamp recordings or calcium imaging, as previously described (Nockemann et al., 2013; Dembla et al., 2017).

### [^35^S]-GTPγS binding experiments

[^35^S]-guanosine-5’-O-(3-thio)-triphosphate experiments were performed on crude membrane fractions of MOR-expressing HEK293 cells to determine mu-agonist-induced G-protein activation (as reflected by the exchange rate of GDP for GTP) at different pH values (6.5–7.4). GTP was replaced by a high concentration of [^35^S]-GTPγS in the assay solvent, and the accumulation of [^35^S]-GTPγS-bound G proteins in the membrane was measured. To this end, membranes were homogenized and dissolved in HEM G-protein buffer at pH 7.4 or 6.5, including freshly added 0.1% (w/v) BSA and 1 mM DTT. In analogy to Ludwig et al. (2003), 50 μg of membrane fractions in duplicates were incubated with GDP (30 μM) and [^35^S]-GTPγS (0.05 nM) for 90 min at 30^°^C to determine dose-response curves and EC_50_ values. Unspecific [^35^S]-GTPγS binding in the presence of non-radioactive GTPγS (10 μM) was subtracted to yield specific binding. Bound and free ligands were separated by rapid filtration under vacuum through Whatman GF/B glass fiber filters soaked in water followed by six washes with Tris Buffer. Bound radioactivity was determined by liquid scintillation spectrophotometry for ^35^S after overnight extraction of the filters in scintillation fluid optiphase HISAFE3. Concentrations of radioactive compound were calculated based on the half-life of ^35^S (87.4 days). Experiments were randomized to compensate for position effects in the filter apparatus or unequal sample processing times. Data processing and analysis were blinded for pH values (6.5 or 7.4) with the help of a colleague.

### Patch clamp experiments

Patch clamp experiments were performed on rat DRGs 24–48 h after their dissection, following a modified protocol from Walwyn et al. (2007). First, cell viability was evaluated by an automated cell counter (Luna, Villeneuve, France) using acridine orange/propidium iodide dyes. During patch clamp recordings, cells were superfused with ECS buffer and visualized using a Zeiss Axiovert 200 inverse microscope (Zeiss, Jena, Germany). Patch clamp pipettes (resistance 3.5–8 MΩ) were produced using the Sutter P-97 puller (Sutter Instruments, Novato, CA, USA) from Borosilicate glass capillaries (Harvard Bioscience, MA, USA). Before the experiment, the glass pipettes were filled with an ICS buffer. Currents were amplified by an EPC-10 patch amplifier and recorded using Pulse software (HEKA, Lambrecht, Germany). Using a pressurized application system (Perfusion Pressure Kit VPP-6; Warner Instruments, Hamden, CT, USA), ECS buffer was added in a steady flow of 800–1,000 μl/min. Neurons with 20–30 μm diameter were selected. After reaching the “giga-seal” at -60 mV, the membrane patch was breached to achieve the whole-cell configuration. The currents were initially recorded at a holding potential of -80 mV in ECS buffer in the absence of test compounds. Subsequently, the cells were depolarized to +10 mV (for 100 ms) for eight times following 20 s intervals. During the first five cycles, only ECS buffer continued to flow. On the sixth cycle, an opioid agonist (fentanyl, NFEPP) was added to the solution. During the last two cycles, the opioid antagonist naloxone was used to block/confirm opioid receptor-mediated effects. Neurons were selected for analysis after the 8th cycle. For experiments using PTX or gallein, the blockers were added to culture media overnight before the day of the experiment (Lu and Ikeda, 2016). Opioid ligands were administered via a perfusion valve system (VC-6; Warner Instruments, Hamden, CT, USA). All recordings were performed at room temperature.

### Calcium imaging

Intracellular calcium imaging experiments were performed as previously described (Dembla et al., 2017). Briefly, cultured rat DRGs were incubated at room temperature with 5 μM Fura 2 AM: (Tocris, Wiesbaden-Nordenstadt, Germany) (1 mM stock in DMSO +0.02% pluronic F-127) for 30 min in growth medium. Following the application of Fura 2 AM, cover slips were transferred to a closed recording chamber (Warner Instruments, Hamden, CT, USA) and continuously perfused with the extracellular solution for 5 min. Fluorescence was monitored every 2 s and images were taken with the DS-Qi1 (Nikon, Tokyo, Japan) at 510 nm wavelength. Alternate excitation at 340 and 380 nm wavelengths were filmed using a Polychrome V monochromator, mounted on a Nikon TE2000 inverted microscope (with 20x Sfluor objective; N.A. 0.5). After a baseline period of ca. 2 min, ligands (as shown in figures) were superfused onto the cells, using a gravity-driven perfusion system, which was controlled by ALA VC3 8 valve control system. Fluorescence intensities of ratio images (340/380 nm) on regions of interest (ROI) (including the whole cell) were quantified by NIKON NIS-Elements software after subtraction of background. To distinguish between neuronal and non-neuronal cells, a high potassium (high K^+^) solution was used to depolarize the cells at the end of the superfusion protocol.

### MOR phosphorylation experiments

Human embryonic kidney (HEK293) cells stably expressing MOR were cultivated at 37^°^C, 5% CO_2_ in Dulbecco’s Modified Eagle’s Medium (DMEM), supplemented with 10% (v/v) fetal bovine serum (FBS), 1% (v/v) penicillin/streptomycin and 1% (v/v) glutamine (Capricorn Scientific, Ebsdorfergrund, Germany). Cells were seeded into poly-L-lysine-coated 60 mm culture dishes and grown until a confluency of 95% was reached. MOR agonists were diluted in PBS buffer of different pH values ranging from 6.0 to 8.0 and were carefully added onto the cell layer for 10 min at room temperature. After the agonist solution was removed, cells were washed with Dulbecco’s PBS, with Ca^2+^ and Mg^2+^ (Capricorn Scientific, Ebsdorfergrund, Germany) and lysed with a detergent buffer (150 mM NaCl; 50 mM Tris-HCl, pH 7.4; 5 mM EDTA; 1% Igepal CA-360; 0.5% deoxycholic acid; and 0.1% SDS) containing phosphatase and protease inhibitors (PhosSTOP and cOmplete mini tablets; Roche, Mannheim, Germany). Cell lysates were then centrifuged at 21,000 × g and 4^°^C for 30 min to obtain the supernatant which was mixed with HA epitope tag antibody agarose conjugates (Thermo Fisher Scientific, Waltham, MA, USA). This mixture was incubated for 2 h at 4^°^C. After washing the agarose beads with detergent buffer for three times, SDS sample buffer (100 mM DTT, 62.5 mM Tris-HCl, 20% glycerol, 2% SDS, and 0.005% bromophenol blue) was added and incubated at 43^°^C for 25 min to release the precipitated receptor from the conjugates.

For electrophoresis, the supernatant was loaded onto an 8% polyacrylamide gel. Subsequently, the proteins were transferred to a PVDF membrane using a semi-dry blotting system (Trans-Blot^§^ Turbo Transfer System, Bio-Rad Laboratories GmbH, Feldkirchen, Germany). Unspecific binding sites were blocked by incubating the membranes in 5% BSA/TBS-T buffer solution for 2 h at room temperature, before the primary antibody diluted in 5% BSA/TBS-T was added overnight at 4^°^C. On the next day, the membrane was washed three times with TBS-T and incubated with the secondary antibody diluted in 5% BSA/TBS-T for 2 h at room temperature. After washing the membrane for another three times, it was placed into SuperSignal West Dura Extended Duration Substrate (Thermo Fisher Scientific, Waltham, MA, USA) to induce a chemiluminescent reaction. The signals were detected using Fusion Fx7 (Peqlab, Erlangen, Germany). In order to reprobe the membrane, bound antibodies were removed by Tris (2-carboxyethyl) phosphine containing stripping buffer.

Western Blot signals were quantified using the software ImageJ. The intensity of each band was calculated by subtracting the background signal from the mean intensity of the selected band area. To determine the magnitude of protein phosphorylation, the ratio of the signal of the phosphorylated protein to the one exhibited by the loading control (total protein concentration) was calculated.

### Data analysis and statistics

Sample sizes were calculated using the G*Power 3.1.2 program with α < 0.05 and a power of 80% based on a defined effect size derived from pilot experiments. Experiments were randomized to compensate for the position effects on plates or filter apparatus and unequal sample processing time. All experiments were performed by an investigator blinded to the sample assignments. The codes were broken after the completion of the experiments. Statistical analyses were performed using GraphPad Prism software (version 5 and 9 for Windows; GraphPad Software Inc., San Diego, CA, USA). Grubbs’ test was performed to identify potential outliers. No samples were excluded from the analysis. All data were assessed for normal distribution and equal variances by D’Agostino and Pearson or Kolmogorov–Smirnov tests. Analysis of concentration-response relationships was performed with simple linear regression using Prism 9 (where y = [^35^S-]GTPγS bound and x = [NFEPP] or [fentanyl]). Ca^2+^ imaging time series of single cells were extracted from stacks of ratio images as averages over the entire cell area in excel sheets, which were then plotted as time vs. ratio 340/380 graphs using Prism 5. For quantitative analyses, the first 10 data points (corresponding to the first 20 s of the experiment) were averaged to form the baseline which was then subtracted from all high potassium (high K^+^) responding cells to obtain Δ ratio 340/380 values. During each recording, we typically were able to record at least from 10 cells, meaning that each trace in Ca^2+^ imaging recordings represents the mean of more than 20 cells. The precise number of cells is stated in the figures or in the figure legends. Data are represented as means ± standard error of the mean (SEM). Detailed statistical analyses are presented in figure legends.

## Results

**FIGURE 1:**
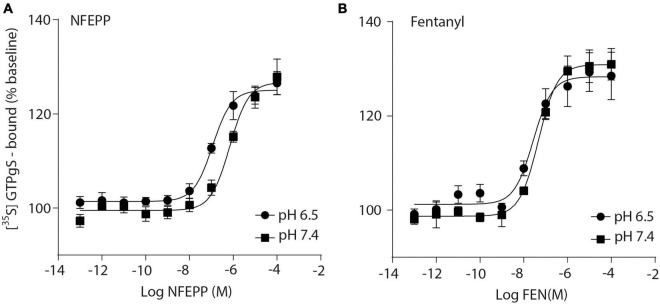
N-(3-fluoro-1-phenethylpiperidine-4-yl)-N-phenyl propionamide (NFEPP) induces significantly higher [^35^S]-guanosine-5’-O-(3-thio)-triphosphate ([^35^S]-GTPγS) binding at low than at normal pH. Dose-dependent [^35^S]-GTPγS binding induced by NFEPP at pH 6.5 (Log EC_50_ = −6.94) and pH 7.4 (Log EC_50_ = −6.16) **(A)**, and by fentanyl at pH 6.5 (Log EC_50_ = −7.57) and 7.4 (Log EC_50_ = −7.33) **(B)**. [^35^S]-GTPγS binding is expressed as percent increase in [^35^S]-GTPγS binding relative to binding in unstimulated samples (*n* = 6 membrane preparations). Data points represent means ± standard error of the mean (SEM) of specific [^35^S]-GTPγS binding in % of baseline. *P* < 0.05 for Log EC_50_ of NFEPP at pH 6.5 vs 7.4, 2-tailed *t*-test.

Initially, we examined whether NFEPP produces higher GTP binding (i.e., MOR activation) at low compared to normal pH. In HEK293 cells stably expressing MOR, both the conventional agonist fentanyl and the pH-dependent agonist NFEPP produced dose-dependent increases in [^35^S]-GTPγS binding (Figure 1). The maximum effects of NFEPP were significantly higher and the EC_50_ values were significantly lower at pH 6.5 than at pH 7.4 (Table 1; *P < 0.05; pH 6.5 versus pH 7.4; *t*-test, *N* = 6 and 7 membranes). In contrast, fentanyl showed similar potencies (EC_50_) and similar maximum effects at both pH values (Figure 1 and Table 1).

**TABLE 1:** EC_50_ values in [^35^S]-guanosine-5’-O-(3-thio)-triphosphate ([^35^S]-GTPγS) binding experiments.

	NFEPP	NFEPP	Fentanyl	Fentanyl
pH	6.5	7.4	6.5	7.4
EC_50_ (mean)	1,340e–007*	7,943e–007	2,905e–008	4,709e–008
Standard error	3,594e–008	2,644e–007	4,103e–009	1,081e–008

*P < 0.05; NFEPP pH 6.5 versus NFEPP pH 7.4; *t*-test, *N* = 6–7.

**FIGURE 2:**
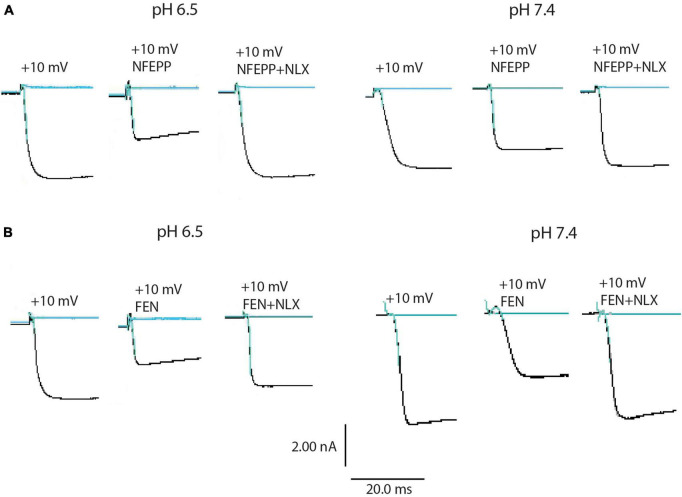
N-(3-fluoro-1-phenethylpiperidine-4-yl)-N-phenyl propionamide (NFEPP) and fentanyl decrease calcium currents. Representative images of inward currents in depolarized (+10 mV/100 ms) isolated rat dorsal root ganglion (DRG) neurons at pH 6.5 and pH 7.4 when treated with NFEPP (100 μM) or NFEPP with naloxone (NLX) (100 μM) **(A)** and with fentanyl (FEN) or FEN (100 μM) with NLX (100 μM) **(B)**. Black traces indicate the inward currents.

**FIGURE 3:**
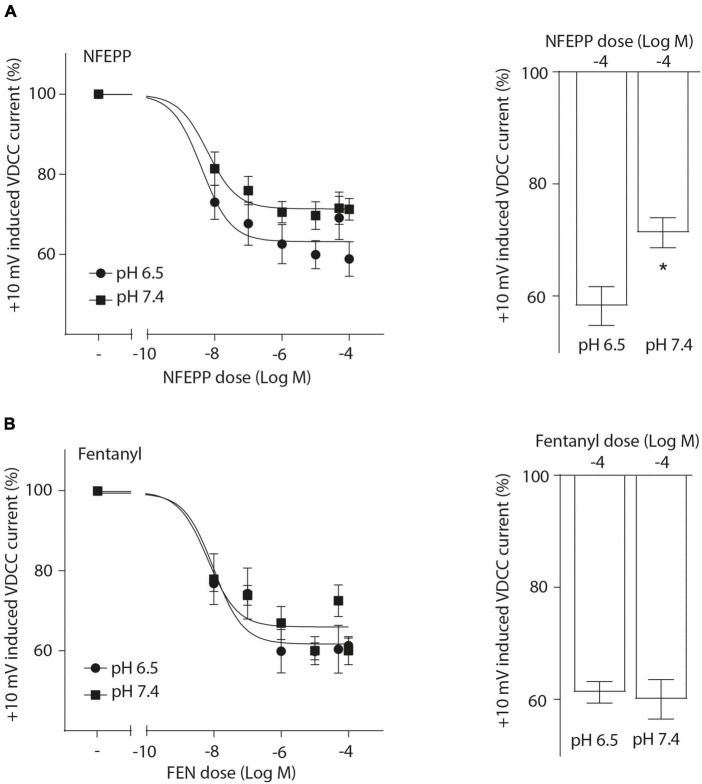
N-(3-fluoro-1-phenethylpiperidine-4-yl)-N-phenyl propionamide (NFEPP) decreases voltage dependent calcium currents more effectively at low than at normal pH. Dose-dependent inhibition of depolarization-induced calcium currents in isolated rat dorsal root ganglion (DRG) neurons by NFEPP at pH 6.5 (Log IC_50_ = −8.02) and pH 7.4 (Log IC_50_ = −7.56) **(A)** or fentanyl at pH 6.5 (Log IC_50_ = −7.89) and pH 7.4 (Log IC_50_ = −7.95) **(B)** (*N* = 14–22) (left). Maximum inhibition by the highest agonist dose (Log M) (right). *P < 0.05 NFEPP at pH 6.5 vs 7.4; two-tailed *t*-test. Data are means ± SEM.

**FIGURE 4:**
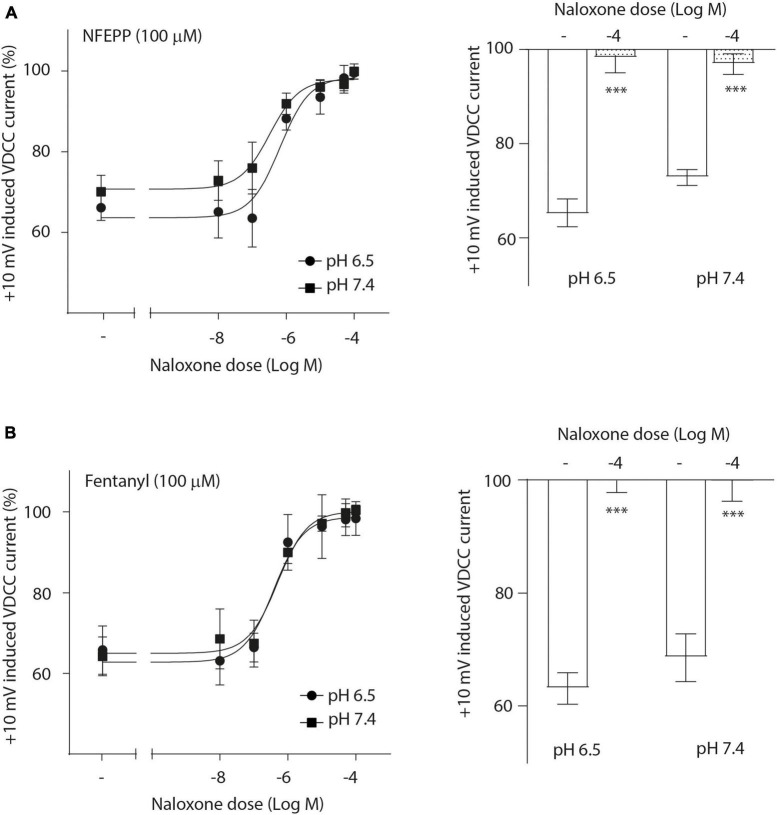
N-(3-fluoro-1-phenethylpiperidine-4-yl)-N-phenyl propionamide (NFEPP)-induced inhibition of calcium currents is mediated by opioid receptors. Dose-dependent effects of naloxone on the maximum inhibition of +10 mV-induced calcium currents by NFEPP **(A)** or fentanyl **(B)** at pH 6.5 and pH 7.4 in isolated rat dorsal root ganglion (DRG) neurons (*N* = 16–20) (left). Effects of the highest naloxone doses (Log M) (right). ***P < 0.001 naloxone vs. control at pH 6.5 vs 7.4; two-tailed *t*-test. Data are means ± SEM.

Next, we assessed the activity of VDCC following NFEPP application at varying pH values in DRG neurons obtained from naive rats. In our experimental set up, the DRG cells initially rested in the whole-cell voltage clamp mode at a constant -80 mV holding potential. When depolarized to +10 mV (100 ms), VDCCs opened to allow Ca^2+^ ions to flow into the cell inducing an inward current. These inward currents were decreased by the application of both NFEPP and fentanyl, and these effects were completely blocked by the opioid antagonist naloxone (Figure 2). Further data analysis was performed to explore the potential impact of NFEPP and fentanyl on the time constants of activation or deactivation of VDCCs. Under our experimental conditions with a sample size of *N* = 14–22, we observed no significant influence of NFEPP or fentanyl on the time constant of VDCC activation. NFEPP treatment dose-dependently decreased VDCC currents at both pH values (6.5 and 7.4) but to different degrees. At pH 6.5, the most effective NFEPP dose (100 μM) attenuated Ca^2+^ currents by 41.8%. This effect was significantly stronger than the effect of NFEPP at pH 7.4 (28.8%) (Figure 3A). In contrast to NFEPP, the dose-response curves upon fentanyl treatment were not different and the most effective fentanyl dose (100 μM) attenuated +10 mV-induced Ca^2+^ currents to the same degree (∼39%) at both pH 7.4 and pH 6.5 (Figure 3B). These findings show that NFEPP is more effective at low pH, whereas the conventional opioid agonist fentanyl is equally effective at both acidic and physiological pH values. Naloxone dose-dependently recovered the +10 mV-induced VDCC currents during both NFEPP and fentanyl treatment (Figure 4). The effects of both NFEPP and fentanyl were completely reversed by the highest naloxone dose (100 μM) at both pH values (Figure 4).

**FIGURE 5:**
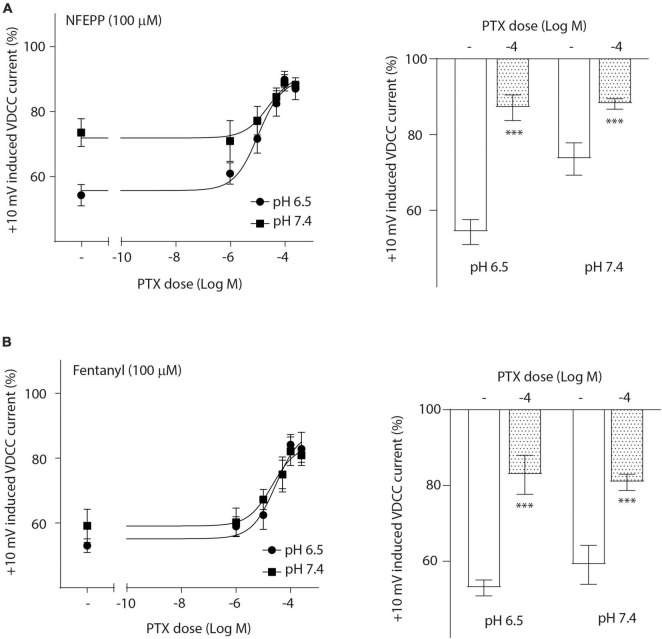
N-(3-fluoro-1-phenethylpiperidine-4-yl)-N-phenyl propionamide (NFEPP)-induced inhibition of calcium currents is G_i/o_-mediated. Dose-dependent effects of pertussis toxin (PTX) on the maximum inhibition of +10 mV-induced calcium currents by NFEPP **(A)** or fentanyl **(B)** at pH 6.5 and pH 7.4 in isolated rat dorsal root ganglion (DRG) neurons (*N* = 17–22) (left). Effects of the highest PTX doses (Log M) (right). ***P < 0.001 PTX vs. control at pH 6.5 vs. 7.4; two-tailed *t*-test. Data are means ± SEM.

**FIGURE 6:**
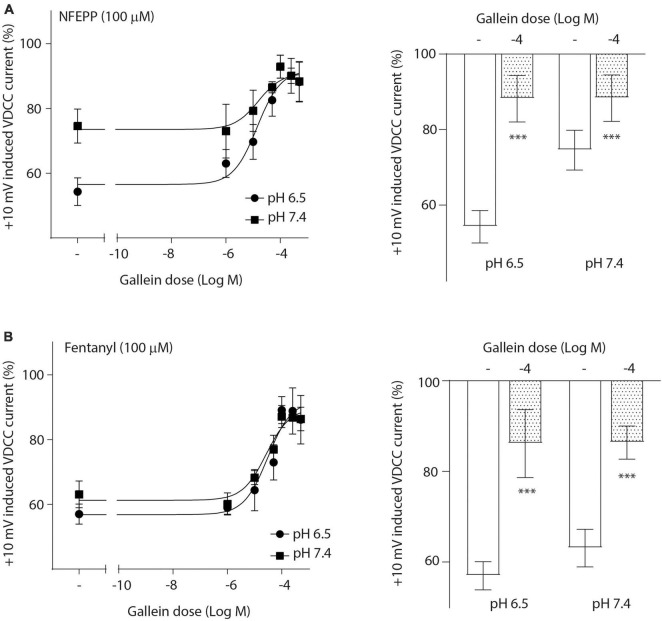
N-(3-fluoro-1-phenethylpiperidine-4-yl)-N-phenyl propionamide (NFEPP)-induced inhibition of calcium currents is dependent on G _βγ_ subunits. Dose-dependent effects of gallein on the maximum inhibition of +10 mV-induced inward calcium currents by NFEPP **(A)** or fentanyl **(B)** at pH 6.5 and pH 7.4 in isolated rat dorsal root ganglion (DRG) neurons (*N* = 15–25) (left). Effects of the highest gallein doses (Log M) (right). ***P < 0.001 gallein vs. control at pH 6.5 vs 7.4; two-tailed *t*-test. Data are means ± SEM.

To investigate the role of G-protein subunits in detail, we initially used PTX, a bacterial toxin which blocks the activity of Gi proteins by modifying the α subunit, preventing them from interacting with downstream effectors and inhibiting their normal signaling pathways, including the subsequent GαGβγ dissociation (Lu and Ikeda, 2016). PTX blocked NFEPP’s effect in a dose-dependent manner (Figure 5A), greatly decreasing the + 10 mV-induced VDCC currents (∼90%) at the highest PTX dose (100 ng/ml). The effect of fentanyl was also blocked (∼83%) in a dose-dependent manner after PTX incubation (Figure 5B). Because PTX does not allow to distinguish between the involvement of Gα versus Gβγ subunits (Lu and Ikeda, 2016), we sought to selectively block Gβγ subunits by gallein (Lu and Ikeda, 2016). Gallein dose-dependently decreased NFEPP-induced effects and recovered +10 mV-induced Ca^2+^ currents at the highest dose at both pH values (∼90%) (Figure 6A). Fentanyl’s effects were also decreased and blocked by the most effective dose of gallein at both pH values (∼88%) (Figure 6B). Together, these results indicate that the effects of both opioid agonists on VDCCs are mediated via activation and subsequent dissociation of heterotrimeric G-protein complexes at both pH values.

**FIGURE 7:**
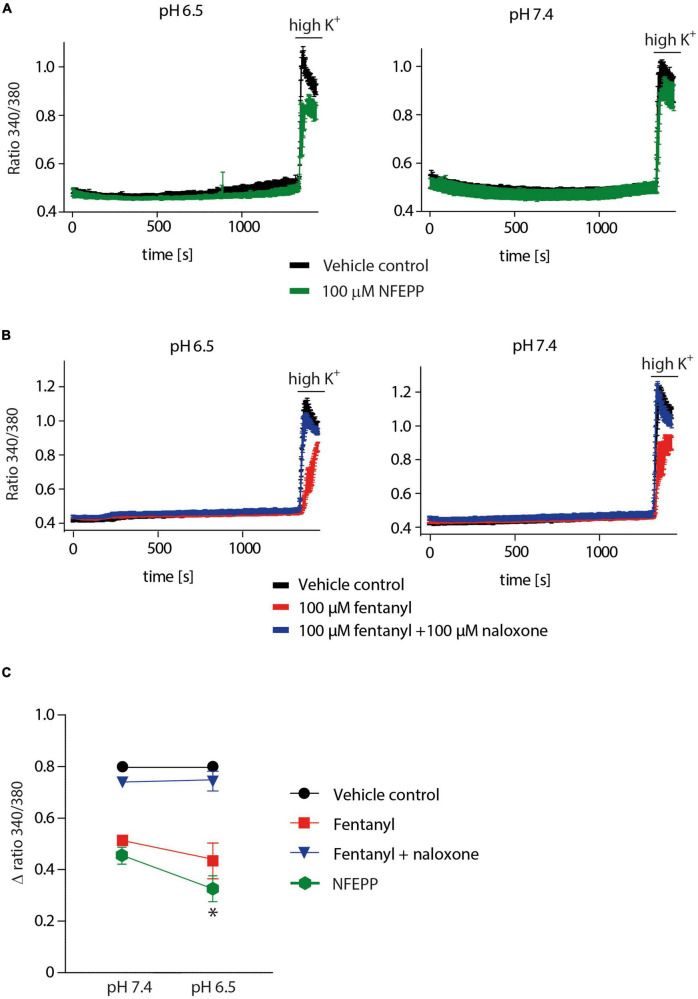
N-(3-fluoro-1-phenethylpiperidine-4-yl)-N-phenyl propionamide (NFEPP) inhibits calcium signals at pH 6.5. **(A)** Fura-2 imaging experiments with rat dorsal root ganglion (DRG) neurons treated with 100 μM NFEPP dissolved in dimethyl sulfoxide (DMSO) (green trace, *N* = 259, nine recordings) and non-treated (vehicle control: 100 μM DMSO) (black trace, *N* = 256, nine recordings) at pH 6.5 and pH 7. **(B)** Calcium responses in rat DRG treated with 100 μM fentanyl dissolved in saline (red trace, *N* = 445, 18 recordings), treated with 100 μM fentanyl +100 μM naloxone (blue trace, *N* = 454, 18 recordings), and non-treated (vehicle control: 0.9% saline) (black trace, *N* = 463, 18 recordings) at pH 6.5 and pH 7. **(C)** Summary, delta ratio 340/360 from **(A,B)** at pH 6.5 and pH 7. *P < 0.05 NFEPP at pH 6.5 vs 7.4; two-tailed *t*-test. Traces represent means ± SEM.

**FIGURE 8:**
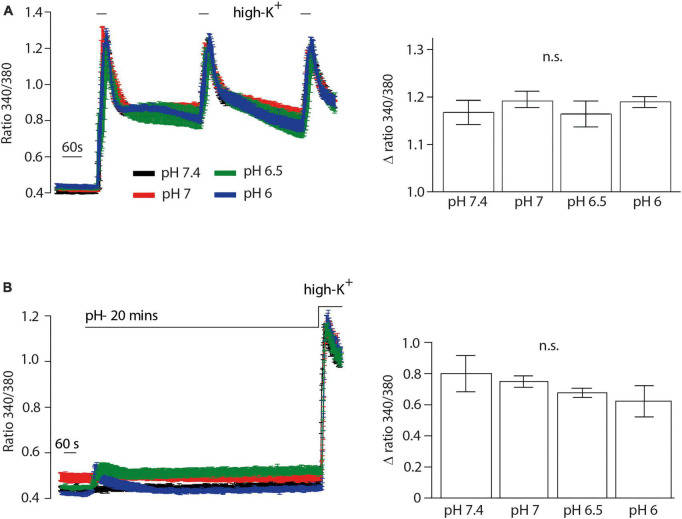
Inflammatory pH conditions *per se* do not affect calcium signals. Fura-2 imaging experiments with rat dorsal root ganglion (DRG) neurons treated with **(A)** short (60 s) and **(B)** long (20 min) application of extracellular pH 7.4 (black trace, *N* = 356), pH 7 (red trace, *N* = 302), pH 6.5 (green trace, *N* = 383) and pH 6 (blue trace, *N* = 386). Twelve recordings for each pH are shown. The right bar graphs represent delta ratio 340/380 at different pH values. *P* > 0.05; One-way ANOVA on ranks. Traces represent means ± SEM.

We then performed Ca^2+^ imaging experiments to further validate our electrophysiological findings. Both NFEPP and fentanyl decreased the Fura Δ ratio 340/380, i.e., the intracellular Ca^2+^ signals were reduced. Naloxone (100 μM) completely reversed the effect of fentanyl (Figures 7B, C). However, in NFEPP-treated neurons, the Ca^2+^ signal was significantly lower at pH 6.5 when compared to pH 7.4 (Figures 7A, C). To investigate whether an acidic microenvironment (as seen in tissue injury) affects VDCC activity, we examined whether extracellular pH alone contributes to the decrease of Ca^2+^ signals. Neither during short (60 s) (Figure 8A) nor long (20 min) (Figure 8B) lasting pH changes, we observed any differences in the Fura Δ ratio 340/380 at either pH 6, 6.5, 7, or 7.4. This indicates that extracellular pH by itself does not influence intracellular Ca^2+^ signals and that NFEPP and fentanyl were solely responsible for the decrease. The control experiments using high potassium indicated that the signals indeed derived from the influx of extracellular Ca^2+^ into neurons (Figure 8).

**FIGURE 9:**
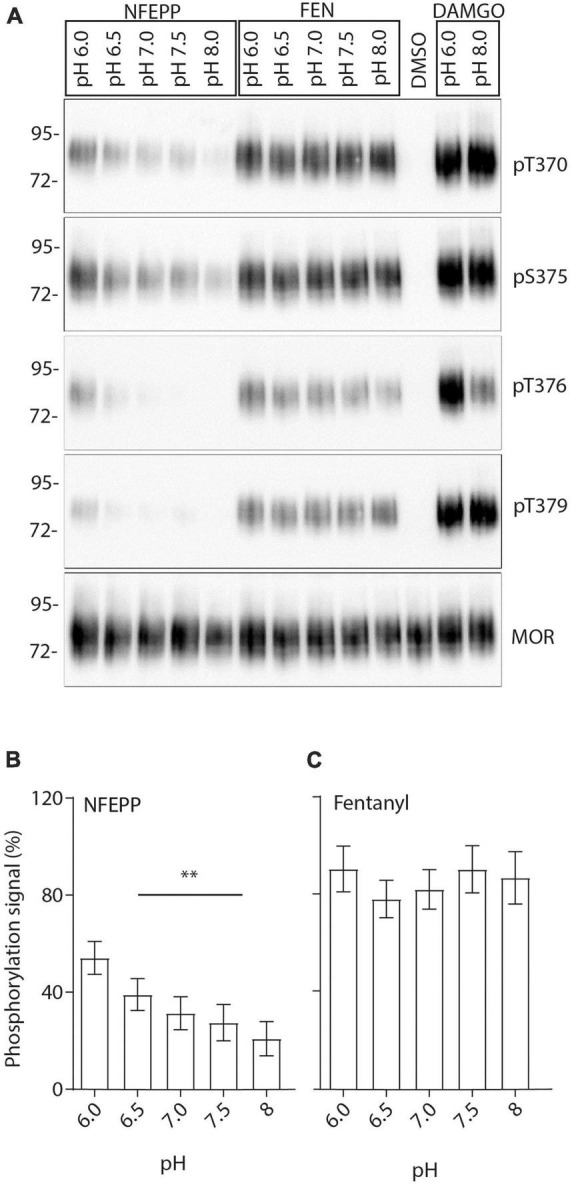
N-(3-fluoro-1-phenethylpiperidine-4-yl)-N-phenyl propionamide (NFEPP)-induced pH-dependent mu-opioid receptors (MOR) phosphorylation in human embryonic kidney (HEK293) cells. **(A)** MOR phosphorylation was analyzed with antibodies specifically reacting with phosphorylated residues T370, S375, T376, and T379. Controls were treated with 1 μM dimethyl sulfoxid (DMSO) (negative control) or 10 μM [D-Ala2, N-MePhe4, Gly-ol]-enkephalin (DAMGO) (positive control). Total phosphorylation signal recorded from residues (T370, S375, T376, T379) when stimulated with 1 μM NFEPP **(B)** or fentanyl **(C)** in a pH-dependent manner (*N* = 16). **P < 0.01; One-way ANOVA on ranks (specific sample pairs are not analyzed). Data are means ± SEM.

Finally, we examined the effects of NFEPP on MOR phosphorylation. MOR expressing HEK293 cells were treated with either NFEPP or fentanyl at pH 6.0–8 (Figure 9A). The strongest NFEPP-induced phosphorylation signal was detected at pH 6.0. The signal intensity diminished with increasing pH values (Figure 9B). In contrast, fentanyl-induced phosphorylation was similar at all pH values (Figure 9C).

## Discussion

Activation of peripheral opioid receptors by peripherally restricted opioid administration was shown to produce strong analgesia in both acute and chronic pain models as well as in patients with arthritis and postoperative pain [reviewed in Wei et al. (2014), Machelska and Celik (2018), Martínez and Abalo (2020)]. Additionally, genetic approaches using conditional opioid receptor knockout in primary afferent nociceptors clearly demonstrated the essential role of peripheral opioid receptors in antinociception following systemic opioid administration (Gaveriaux-Ruff et al., 2011; Weibel et al., 2013; Sun et al., 2019; Jin et al., 2022). Accordingly, the selective activation of peripheral MOR on DRG neurons innervating injured (inflamed) tissues appears to be a promising strategy to inhibit pain while avoiding central side effects. NFEPP is an opioid with a lower dissociation constant (pK_a_) that was shown to bind and activate MORs more effectively at low pH values. Therefore, even though NFEPP is presumably distributed throughout the body (including the CNS) after systemic (intravenous) application, it only activates MORs in peripheral inflamed tissues where the pH is more acidic [reviewed in Stein (2018)]. In this study, we expanded our understanding of NFEPP’s antinociceptive effects by investigating MOR signaling in transfected HEK cells and sensory neurons.

First, we found that NFEPP produced more efficient G-protein activation at low pH, while fentanyl induced comparable G-protein activation at both physiological and low pH values. This is consistent with our previous studies (Spahn et al., 2017, 2018; Meyer et al., 2019). The small difference in EC_50_ values of fentanyl between the present and the previous study by Meyer et al. (2019) could be due to variations in assay protocols, cell cultures or handling by the different experimenters. Considering the standard errors of the mean given in **Table 2** of Meyer et al. (2019), these differences appear not significant. In addition, fentanyl appears slightly more potent than NFEPP in this *in vitro* assay.

Opioid receptor activation can result in blockade of membrane VDCCs, which are essential for the excitation of sensory neurons [reviewed in Machelska and Celik (2018), Weiss and Zamponi (2021)]. To verify that NFEPP acts through the same mechanisms, we examined the inhibition of inward Ca^2+^ currents at both physiological (7.4) and acidic (6.5) pH values. These currents were more efficiently reduced following NFEPP treatment at acidic pH (in contrast to pH 7.4), while fentanyl attenuated Ca^2+^ currents to similar degrees at both pH values. By using naloxone, we confirmed that the effects of both NFEPP and fentanyl were mediated by opioid receptors. These results are consistent with experiments in mouse intestinal neurons that have assessed nociceptor excitability by measuring the rheobase (minimum input current required to fire an action potential) (Jiménez-Vargas et al., 2022). Together, these findings support our previous studies where fentanyl, likely due to its higher pK_a_ value (8.44) and comparable protonation, was equally effective at both pH values, while NFEPP (pK_a_ = 6.82) showed increased activity only at acidic conditions (Spahn et al., 2017, 2018). Interestingly, in our present experiments NFEPP inhibited VDCC also at normal pH, whereas it did not produce analgesia in healthy tissues *in vivo* (Spahn et al., 2017). In this context, it must be remembered that opioid receptor activation modulates not only Ca^2+^ currents but also K^+^-, Na^+^-, TRP-channels, arrestins and many other messengers [reviewed in Machelska and Celik (2018)]. Furthermore, only a fraction of normal DRG neurons express functional opioid receptors but this fraction increases under conditions of peripheral tissue inflammation [reviewed in Machelska and Celik (2018)]. These phenomena, their interactions and their relevance for *in vivo* analgesia at normal versus low pH need to be investigated in future studies.

To examine whether the dissociation of G-protein subunits differs between pH values following NFEPP or fentanyl treatment, we blocked the GDP-GTP exchange at Gαi/o subunits using PTX, and we inhibited Gβγ subunits using gallein in our patch clamp analysis. Gβγ subunits can directly deactivate VDCCs following MOR activation [reviewed in Proft and Weiss (2015)]. In line with this notion, we found that NFEPP-induced inhibition of inward Ca^2+^ currents was dependent on Gβγ subunits. Consistent with its indistinguishable effect on Gα versus Gβγ subunits (Lu and Ikeda, 2016), PTX also blocked Ca^2+^ currents, indicating that, by hindering GDP-GTP exchange, consequent Gαiβγ dissociation is also impaired. Although PTX and gallein significantly reduced the Ca^2+^ currents by 83–90%, they did not achieve complete reversal to 100%. This observation may be attributed to experimental limitations, such as the incubation period with gallein or PTX. Additionally, it is important to consider that these compounds may not fully block VDCCs like a direct VDCC antagonist, as their effects are mediated indirectly through the modulation of G protein signaling.

Further validation of our electrophysiological data was performed using Ca^2+^ imaging experiments. Ca^2+^ signals were only reduced in NFEPP-treated DRG neurons at pH 6.5. Fentanyl, however, significantly reduced Ca^2+^ signals at both pH values. Control experiments using naloxone and high potassium confirmed that the signals were modulated by opioid receptors and derived from influx of Ca^2+^ into neurons, similar to previous studies (Hochstrate et al., 1995). Both calcium imaging (Figure 8) and patch clamp experiments (data not shown) demonstrated that extracellular low pH (6.5) alone did not affect Ca^2+^ signals.

It is important to note that high-threshold VDCCs such as N-type are the major channels associated with pain transduction and that blocking these channels is a common strategy in pain management [Saegusa et al., 2001, reviewed in Castro-Junior et al. (2018)]. In addition, low-threshold VDCCs such as T-type and L-type have been implicated but the significance of these channels in primary afferent nociceptors is not well-defined. While it is widely recognized that, following MOR activation, Gβγ subunits can decrease the overall conductance of high threshold VDCCs, there is only limited evidence to suggest that Gβγ subunits can block low threshold VDCCs [reviewed in Castro-Junior et al. (2018)]. Taken together, our data are consistent with the notion that VDCCs in primary afferent neurons play an important role but are not the only molecules that determine *in vivo* analgesic effects following the activation of peripheral opioid receptors.

Finally, the ability of NFEPP to phosphorylate MOR was examined under different pH conditions. Phosphorylation of MOR at residues T370, S375, T376, and T379 has been shown to play a crucial role in receptor desensitization, binding of arrestins and internalization (Mann et al., 2015). Our western blot analysis showed that NFEPP induced the strongest MOR phosphorylation at pH 6.0. The signal was most pronounced at MOR residues T370 and S375 and decreased with increasing pH levels. In comparison, fentanyl caused similar phosphorylation signals of MOR regardless of increasing pH values. Notably, the antibodies used in our experiments only detect intracellular phosphorylation sites at the C-terminal of MOR (Mann et al., 2015). Assuming that non-specific pH effects would produce similar phosphorylation signals for all pH values and both agonists, our findings strongly suggest that the differing signals produced by NFEPP were due to extracellular pH changes. Importantly, fentanyl apparently stimulates MOR independent of extracellular pH, while NFEPP activates MOR preferentially at low pH values.

In conclusion, we have now demonstrated that the selective peripheral activity of NFEPP under inflammatory conditions is not only dependent on enhanced (extracellular) binding of NFEPP to MOR at low pH, but that these events translate into enhanced (intracellular) G-protein activation, subsequent inhibition of VDCC and MOR phosphorylation. Our results show that during both NFEPP and fentanyl treatment, VDCCs are directly modulated by Gβγ subunits dissociated from Gαi/o. Importantly, MOR signaling induced by NFEPP is more effective at low pH. Given the known decrease of extracellular pH in injured tissues (Stein, 2018) and the co-expression of MOR and VDCC in sensory neurons (Proft and Weiss, 2015), our study uncovers the neural mechanisms underlying the antinociceptive effects of NFEPP in injured tissues. We cannot exclude that NFEPP-induced MOR activation also differentially influences other ion channels (e.g., TRP, Na^+^, K^+^) or intracellular messengers (e.g., arrestins) in nociceptive neurons. This remains to be elucidated in future work.

## Data availability statement

The raw data supporting the conclusions of this article will be made available by the authors, without undue reservation.

## Ethics statement

This animal study was reviewed and approved by Landesamt für Gesundheit und Soziales, Berlin, Germany.

## Author contributions

CS, MC, VS, and SS designed the experiments. MC, VS, NB, and SD performed the experiments and analyzed the data. All authors contributed to writing the manuscript and approved the final version of the manuscript.
